# Effects of prenatal professional breastfeeding education for the family

**DOI:** 10.1038/s41598-022-09586-y

**Published:** 2022-04-02

**Authors:** Haifeng Gao, Jie Wang, Jing An, Shuyu Liu, Yan Li, Songtao Ding, Yi Zhang, Ying Chen

**Affiliations:** 1 Breast Disease Prevention and Treatment Centre, Haidian Maternal and Child Health Hospital, No. 53 Suzhou Street, Haidian District, Beijing, 100080 China; 2 Breast Centre, Amcare Women’s and Children’s Hospital, Beijing, China

**Keywords:** Diseases, Health care, Risk factors

## Abstract

Mastering the correct breastfeeding posture remains a challenge for many new mothers. Generalized pregnancy breastfeeding education plays a role in helping mothers master breastfeeding positions and prevent nipple damage. This study prospectively analyzed the effects of prenatal professional breastfeeding education for the family on mastering the lactation latch and preventing nipple damage. Medical records of pregnant women in the authors' hospital from April 2020 to July 2020 were prospectively analyzed. A total of 342 patients were enrolled and divided into experimental and control groups according to whether or not they had received prenatal professional breastfeeding education for the family by the random number table method. The difference in the mastery rate of the postpartum breastfeeding posture and nipple damage was examined three days postpartum. The mastery rate in the experimental group (88.5%) was significantly higher than that in the control group (63.8%), whereas the rate of nipple damage in the experimental group (23.1%) was significantly lower than that in the control group (46.9%). Prenatal professional breastfeeding education for the family can promote mothers' mastery of breastfeeding latch skills and reduce the risk of nipple damage.

## Introduction

Exclusive breastfeeding has been reportedly beneficial for the child's mortality, health, and development^1^. As such, the World Health Organization recommends exclusive breastfeeding as the optimal form of feeding for infants aged up to 6 months^2^. Although most women start breastfeeding immediately after delivery, difficulties often arise, such as nipple damage, which is one of the primary problems^3^, with approximately 80–90% of breastfeeding women complaining of nipple pain and fissures^4^. However, some nipple injuries are still unresolved after removing the cause; chronic nipple wounds require debridement^5^, and persistent nipple pain is associated with a low breastfeeding rate at six months postpartum. Nipple damage was found to be significantly associated with lactational mastitis^6^ and the most common risk factor for mastitis^7^.

To prevent premature cessation of breastfeeding, nipple damage should be prevented in new mothers. Nipple pain and damage may occur due to inappropriate positioning and latching (72.3%)^8^. Evidence suggests that early postnatal education on the positioning and attachment technique, early observation of mothers, and correcting breastfeeding techniques at an early stage may reduce nipple trauma^9^. Adequate breastfeeding support, including evaluation of latching, positioning, and feeding at the breast, could prevent nipple cracks^10^. More prenatal education or counseling has been used in reducing breastfeeding complications^11^. A one-to-one breastfeeding training more effectively prevented nipple cracks^12^.

The authors prospectively selected pregnant women and their families for analyzing the difference in the percentage of women who had mastered the breastfeeding posture and incidence of nipple damage at three days postpartum between mothers who received prenatal professional family breastfeeding educational training and those who did not receive professional guidance.

## Methods

### Patients

Medical records of all pregnant women at the Haidian Maternal and Child Health Hospital from April 2020 to July 2020 were reviewed. Patients who met the following criteria were proposed and collected: (1) primiparous women documented in our hospital and ready for delivery, (2) those aged between 25 and 40 years, (3) those with the age of gestation of ≥ 32 weeks counting from the first day of the last menstrual, (4) those who understood the research objective and signed the informed consent. However, (1) patients with a history of breast surgery; (2) who suffer from diseases that contraindicated breastfeeding; (3) with endocrine-related diseases, such as pregnancy hypertension, gestational diabetes, and hypothyroidism; (4) with pregnancy in vitro fertilization; (5) the pregnant women and families with a plan to abandon breastfeeding for various reasons were excluded. Incomplete data or lost respondents were considered as drop-outs and lost to follow-ups.

Patients were randomly assigned to one of two groups by the random number table method: one (the experimental group) receiving prenatal professional breastfeeding education for the family, and the other (the control group) receiving a normal breastfeeding course. Researchers performed follow-up examinations on the third day after delivery in the hospital, assessed and analyzed the mastery of breastfeeding posture, and examined any nipple damage in both groups. In total, 342 participants were finally enrolled in the study.

### Sample size calculation

Approximately 80–90% (85%) of breastfeeding women had experienced nipple pain and fissures, Through the implementation of this study, The rate of nipple pain and fissures is expected to reach 60%. PASS11 were used to measure the sample size, α-0.05, β = 0.1 and the following results were obtained: A sample size of 50 achieves 100% power to detect a difference (P1–P0) of 0.1500 using a two-sided binomial test. The target significance level is 0.0500. The actual significance level achieved by this test is 0.0274. These results assume that the population proportion under the null hypothesis is 0.8500. The sample size was adequate.

### Data collection

The following data were collected for all pregnant women on the third day after delivery in hospital: demographics, age, delivery age of gestation in weeks, delivery mode, baby's sex, the mastery of the breastfeeding posture, and nipple damage were evaluated by the nurse through checking the mother's nipples and observing the breastfeeding process.

The standard of mastery of breastfeeding posture was defined as the following: the mother adjusts the breastfeeding posture independently without any assistance, either positioning herself or her infant to get the infant latched onto the breast^13^.

The standard criteria for nipple damage included the following: no nipple trauma present on mother's nipples, including redness, blistering, bruising, bleeding, or scabbing^13^.

This randomized controlled trial was approved by the medical ethics committee of the Haidian District Center for Disease Control and Prevention of Beijing, and informed consent for the study participation was obtained. In addition, all methods were conducted according to the relevant guidelines and regulations.

### Breastfeeding education

In the experimental group, all pregnant women and their families studied ten online prenatal professional breastfeeding courses at any time between 32 weeks gestation and delivery. The courses used the mobile application named "lizhiweike," which is affiliated with Shenzhen Shifang Ronghai Technology Co., LTD., it is a free online education platform. The course was presented by health professionals trained in the IBCLC core curriculum, including two nurses (one IBCLC) and four doctors (two IBCLC) , and each course lasted for 30 min. The following contents were included: (1) the importance of breastfeeding for the mother and child, (2) the influence of abnormal breast development on lactation and lactation skills, (3) prevention strategies for postpartum milk swelling and milk deposition, (4) prevention and nursing guidance for nipple damage, (5) self-coping methods of too much or too little breast milk, (6) prevention and treatment of lactation mastitis and mammary abscess, (7) reasonable selection of drugs during lactation, (8) breastfeeding during the separation of mother and child, (9) how to grasp the golden period of breastfeeding within 24-h postpartum, and (10) the timing and method of weaning.

In the control group, all pregnant women only received a breastfeeding course of up to 30 min at the pregnant woman's school in the hospital taught by a doctor (including the benefits of breastfeeding and possible breastfeeding problems and related causes).

### Data analysis

The chi-squared test was used to compare differences in the mastery rate of the postpartum breastfeeding posture and the incidence of nipple damage at three days postpartum between the two groups. The overall significance level was set to an alpha of 0.05. The statistical analysis was conducted using the Statistical Package for the Social Sciences Statistics software version 24.0 (IBM, Chicago, IL, USA).

## Results

### General information and clinical characteristics of patients

Table 1 shows the patients' demographics and clinical characteristics.

**Table 1:** General information and clinical characteristics of patients.

General information and clinical characteristics	Experimental group	Control group	p*
Age (years; min–max)	31.26 ± 4.22	31.13 ± 3.98	0.447*
Gestational age (weeks; min–max)	38.75 ± 1.09	38.92 ± 1.04	0.910*
Female (n, %)	85	77	0.452*
Male (n, %)	87	83	
Vaginal delivery (n, %)	118	103	0.929*
Cesarean delivery (n, %)	64	57	

Values are given as mean ± SD unless otherwise specified.

*P-value of < 0.05 was considered statistically significant. Onset time after production.

### Comparison of postpartum breastfeeding posture mastery

The experimental group consisted of 182 pregnant women, comprising 161 (88.5%) women who mastered the breastfeeding posture and 21 (11.5%) women who did not master the posture. In the control group, 102 (63.8%) women mastered the breastfeeding posture and 58 (36.3%) women did not master the posture (χ^2^ = 29.271; *P* < 0.001) (Table 2).

**Table 2:** Comparison of the mastery of the postpartum breastfeeding posture and the incidence of nipple damage.

Result	Experimental group	Control group	χ^2^	P*value
Mastered	161 (88.5%)	102 (63.8%)	29.271	< 0.001*
Not mastered	21 (11.5%)	58 (36.3%)		
Nipple damage	42 (23.1%)	75 (46.9%)	21.426	< 0.001*
No nipple damage	140 (76.9%)	85 (53.0%)		

*P-value < 0.05 was considered statistically significant.

### Comparison of the incidence of nipple damage

In the experimental group, 42 (23.1%) pregnant women had nipple damage and 140 (76.9%) women had no nipple damage. In the control group, 75 (46.9%) pregnant women experienced nipple damage and 85 (53.1%) did not (χ^2^ = 21.426; *P* < 0.001) (Table 2).

## Discussion

Postpartum mothers are prone to incorrect breastfeeding posture, which can easily cause nipple damage. Lack of breastfeeding knowledge and skill can result in difficulties in supporting breastfeeding mothers to effectively identify and correct the cause of nipple damage and improve the lactation latch as soon as possible. Prenatal breastfeeding education has been reported as a factor that influences breastfeeding^14^. Prenatal breastfeeding counseling can increase mothers' breastfeeding self-efficacy and solves most breastfeeding problems during the postpartum period^15^. Research on breastfeeding education shows that breastfeeding mothers might benefit from specifically trained health professionals and IBCLCs^16,17^. Intervention targeting fathers at antenatal periods may positively influence the breastfeeding practices of mothers^18^.

The significance of prenatal professional breastfeeding education is to convey the concept of breastfeeding to pregnant women and their families. Pregnant women need to understand the lactation problems they may encounter during lactation and their causes. They need to receive training on correct and feasible self-treatment skills to effectively prevent breastfeeding problems and deal with them with confidence. This study provides prenatal professional breastfeeding education for the family. The mastery rate of lactation latching skills was better, and the incidence of nipple damage was lower in the experimental group than in the control group. This illustrates that prenatal professional breastfeeding education can help pregnant women master the breastfeeding posture and reduce the incidence of nipple damage.

This study has several limitations. First, no subgroup analysis was conducted on the pregnant breast development (e.g., nipple retraction) and oral development of newborns. Second, lactation skills and nipple injury also require long-term follow-up. Third, this study conducted no further follow-up on the breastfeeding duration and exclusive breastfeeding rate.

In conclusion, this study revealed that prenatal professional breastfeeding education for pregnant women and their families plays a role in mastering breastfeeding latch skills and reducing nipple injury.

## Data Availability

The datasets generated and analyzed in this study are not publicly available because the study involved the patients' breasts, which are private body parts; these datasets can be made available by the corresponding author upon reasonable request.

## References

[1] Victora CG (2016). Breastfeeding in the 21st century: epidemiology, mechanisms, and lifelong effect. Lancet.

[2] WHO. Global strategy for infant and young child feeding. Geneva: World Health Organization (2002).

[3] Spencer JP (2008). Management of mastitis in breastfeeding women. Am Fam Physician..

[4] Niazi A (2018). A systematic review on prevention and treatment of nipple pain and fissure: are they curable?. J Pharmacopuncture..

[5] Gao, H. *et al*. A retrospective analysis of debridement in the treatment of chronic injury of lactating nipples. *Sci. Rep. ***11,** 3625; 10.1038/s41598-021-83172-6 (2021). 10.1038/s41598-021-83172-6 PMC7878889 33574449

[6] Deng, Y. *et al*. Maternal risk factors for lactation mastitis: a meta-analysis*. West. J. Nurs. Res.* **43,** 698–708 (2021). 10.1177/0193945920967674 33089754

[7] Wilson E, Woodd SL, Benova L (2020). Incidence of and risk factors for lactational mastitis: a systematic review. J Hum Lact..

[8] Puapornpong P, Paritakul P, Suksamarnwong M, Srisuwan S, Ketsuwan S (2017). Nipple pain incidence, the predisposing factors, the recovery period after care management, and the exclusive breastfeeding outcome. Breastfeed Med..

[9] Oliveira, F. S., Vieira, F., Guimarães, J. V., Aredes, N. D., & Campbell, S. H. Lanolin and prenatal health education for prevention of nipple pain and trauma: randomized clinical trial. Lanolina y educación para la salud en la prevención de dolor y lesiones en los pezones: ensayo clínico aleatorizado. *Enferm Clin (Engl ed).* **31,** 82–90 (2021). 10.1016/j.enfcli.2020.10.030 33277168

[10] Gianni ML (2019). Breastfeeding difficulties and risk for early breastfeeding cessation. Nutrients.

[11] Karaçam Z, Sağlık M (2018). Breastfeeding problems and interventions performed on problems: systematic review based on studies made in Turkey. Turk Pediatri Ars..

[12] Eksioglu A, Yesil Y, Demir Gungor D, Ceber Turfan E (2017). The effects of different breastfeeding training techniques given for primiparous mothers before discharge on the incidence of cracked nipples. Breastfeed Med..

[13] Johnson TS, Mulder PJ, Strube K (2007). Mother-Infant Breastfeeding progress tool: a guide for education and support of the breastfeeding dyad. J Obstet Gynecol Neonatal Nurs..

[14] Sayres S, Visentin L (2018). Breastfeeding: uncovering barriers and offering solutions. Curr Opin Pediatr..

[15] Shafaei FS, Mirghafourvand M, Havizari S (2020). The effect of prenatal counseling on breastfeeding self-efficacy and frequency of breastfeeding problems in mothers with previous unsuccessful breastfeeding: a randomized controlled clinical trial. BMC Womens Health.

[16] Haase B, Brennan E, Wagner CL (2019). Effectiveness of the IBCLC: have we made an impact on the care of breastfeeding families over the past decade?. J Hum Lact..

[17] Navarro, I., Soriano, J. M., & Laredo, S. Applying systematic review search methods to the grey literature: a review of education and training courses on breastfeeding support for health professionals. *Int. Breastfeed J*. **16**,31(2021). 10.1186/s13006-021-00373-5 PMC8025331 33823895

[18] Bich, T. H., Long, T. K., & Hoa, D. P. Community-based father education intervention on breastfeeding practice: results of a quasi-experimental study. *Matern. Child. Nutr.* **15,** e12705 (2019). 10.1111/mcn.12705 PMC7199053 30748110

